# A Laboratory Guide in Urinalysis and Toxicology

**Published:** 1887-03

**Authors:** 


A Laboratory Guide in Urinalysis and Toxicology. *By* R. A. Witthaus, A.M., M.D. *Pp. 73. New York:* William Wood & Company. *1886.*

Professor Witthaus gives us here a very useful little manual for laboratory work.

The descriptions of methods and tests are accurate and concise, as one would naturally expect from an author who has already made himself known by numerous contributions to medical chemistry.

The work is full enough to satisfy the wants of the general medical student.

A blank page is left opposite each printed page for notes.

